# Identity Selection and the Social Construction of Birthdays

**DOI:** 10.3389/fpsyg.2021.693776

**Published:** 2021-10-26

**Authors:** Brett W. Pelham, Tracy DeHart, Mitsuru Shimizu, Curtis D. Hardin, H. Anna Han, William von Hippel

**Affiliations:** ^1^Montgomery College, Germantown, MD, United States; ^2^Loyola University Chicago, Chicago, IL, United States; ^3^Department of Psychology, Southern Illinois University Edwardsville, Edwardsville, IL, United States; ^4^Department of Psychology, Brooklyn College and Graduate Center, City University of New York, Brooklyn, NY, United States; ^5^National Institutes of Health, Bethesda, MD, United States; ^6^School of Psychology, University of Queensland, St. Lucia, QLD, Australia

**Keywords:** self, magical thinking, basking in reflected glory, identity, superstition, birth, holidays, birthdays

## Abstract

We argue that rather than being a wholly random event, birthdays are sometimes selected by parents. We further argue that such effects have changed over time and are the result of important psychological processes. Long ago, U.S. American parents greatly overclaimed holidays as their children's birthdays. These effects were larger for more important holidays, and they grew smaller as births moved to hospitals and became officially documented. These effects were exaggerated for ethnic groups that deeply valued specific holidays. Parents also overclaimed well-liked calendar days and avoided disliked calendar days as their children's birthdays. However, after birthday selection effects virtually disappeared in the 1950s and 1960s, they reappeared after the emergence of labor induction and planned cesarean birth. For example, there are many fewer modern U.S. births than would be expected on Christmas Day. In addition, modern parents appear to use birth medicalization to avoid undesirable birthdays (Friday the 13th). We argue that basking in reflect glory, ethnic identity processes, and superstitions such as magical thinking all play a role in birthday selection effects. Discussion focuses on the power of social identity in day-to-day judgment and decision-making.

## Introduction

In the United States today, about 98% of births take place at hospitals. The exact timing of such births is very carefully documented. But in 1900, virtually all U.S. births took place at home, often without any formal medical supervision (MacDorman et al., 2012, 2014). For many U.S. parents in 1900, getting a birth certificate took time. This meant that there was some wiggle room in the assignment of children's dates of birth. Where there is wiggle room, there is often wiggling, and such wiggling might reflect motivated social cognition. For example, parents might wish to claim a positive social identity for their child—or avoid a birthday associated with tragedy. Any such birthday selection effects might have decreased over time, as births became better documented. On the other hand, modern birth technologies now give some parents some choice about the exact day on which their child is born. The goals of the current paper are to (a) examine birthday selection effects (including how they have changed over time) and (b) examine some of the likely drivers of such effects.

### The Birthday Selection Effect

If birthday selection effects do exist, parents should probably be expected to prefer well-liked days, such as holidays. In the U.S., people have long celebrated major holidays with time off from work and with family gatherings (e.g., Christmas Day, New Year's Day, and U.S. Independence Day). In addition, there are minor and informal holidays such as Abraham Lincoln's birthday (February 12), Valentine's Day (February 14, the “day of love”), and St. Patrick's Day (March 17). (For readers not familiar with the ten U.S. holidays examined here, we provide summaries in Appendix A). In addition to preferring holidays for the birth of their child, parents might also prefer days that simply have positive associations. The number 1, for example, is strongly associated with status and success.

Parents might also wish to avoid certain birthdays for their children. It is possible, for example, that some parents might wish to avoid a Halloween (October 31) birthday. Because the number 13 is widely considered unlucky, many parents might wish to avoid this date. It stands to reason that this aversion might be even stronger than usual for *Friday* the 13th—which is directly associated with the betrayal and crucifixion of Jesus. Finally, it would not be surprising if—shortly after the infamous 9/11 attacks, many U.S. parents wished to avoid giving their child a September 11 birthday.

### Explanations for the Birthday Selection Effect

The second goal of this work is to see what psychological factors influence birthday selection effects. In the following section, we review theoretical and practical explanations for the birthday selection effect. Basking in reflected glory, identity processes, superstition (magical thinking), and hospital staffing decisions may all play a role in birthday selection effects.

People often strive to claim positive social identities (Conway and Ross, 1984; Steele et al., 1993; Aronson et al., 1995; Sherman and Kim, 2005). Consider research on basking in reflected glory. The same students who report that “we won” after their college team wins a game more often report that “they lost” after their college team loses (Hastorf and Cantril, 1954; Cialdini et al., 1976; Kunda, 1990; and see also Balcetis and Dunning, 2006). Claiming positive identities is often highly symbolic, such as liking someone who shares our first name—or noting that we share a birthday with a famous person (Burger et al., 2004; Jones et al., 2004; Sherman et al., 2009; Townsend and Sood, 2012). Therefore, parents may want their child to share a birthday with Lincoln, Jesus, or St. Patrick as a way to bask in the glory of said famous person.

The desire to claim a positive social identity for one's child might influence parents' judgments about and memories of their children (DeHart et al., 2004, 2006, 2011; Wenger and Fowers, 2008). When most births took place at home, parents may have had difficulty remembering whether their child was born on a holiday vs. in close proximity to it. In such an era, there should have been a nudge toward recalling the holiday. Further, in the present as well as in the past, more beloved holidays would presumably be more strongly preferred than less beloved holidays. A core idea behind shared reality theory (Hardin and Higgins, 1996) is that things feel true and right to the degree that we think other people believe in them. Thus, according to the prevailing cultural beliefs of early twentieth century America, sharing a birthday with Jesus should be even more desirable than sharing a birthday with Saint Patrick.

However, basking in the reflected glory of sharing a birthday with Jesus (Christmas Day) might disappear—or even reverse—in modern samples. Consider how Christmas has changed in the past 120 years. In 1900, Christmas was celebrated modestly, with only one in five Americans putting up Christmas trees (Restad, 1995). Over time, Christmas took on much greater significance. Similar cases can be made for other major holidays. Once major holidays increased in cultural importance, parents may have realized that others would be too focused on the holiday itself to focus directly on those connected to it. It may be hard to bask in the reflected glory of something that it is blinding. Thus, as Christmas became the center of so much collective attention in the past few decades, many parents may have realized that giving birth on this day had disadvantages as well as advantages. Further, even if modern parents still prefer this day of birth, hospital staff and administrators might wish to avoid it (so that more staff members could have this day off from work).

In the United States, one of the most important aspects of social identity has long been ethnicity (Hutnik, 1991; Phinney and Devich-Navarro, 1997; Twenge and Crocker, 2002). The importance of holidays varies greatly across ethnic groups. St. Patrick's Day is a minor and unofficial holiday in the United States, but it is an official public holiday in Ireland. Along similar lines, most Americans are familiar with *Cinco de Mayo* (which commemorates a May 5 Mexican military victory over French invaders). But among Chicanos, *Cinco de Mayo* has much greater cultural significance. If birthday selection is magnified for holidays that matter more in a person's subculture, there should be (a) a larger birthday selection effect for St. Patrick's Day for Irish immigrants and (b) a larger birthday selection effect for *Cinco de Mayo* for Mexican immigrants.

Superstitions and other forms of “magical thinking” may also contribute to birthday selection effects. Most parents clearly want others to form positive impressions of their children. But independent of any such concerns, parents *themselves* may prefer to give birth to children on some days more than others. Research on the representativeness heuristic suggests that people assume that when two things share surface features, they share deeper features. Thus, teams wearing black jerseys are judged to be more aggressive than teams wearing any other color of jersey (Kahneman and Tversky, 1972; Frank and Gilovich, 1988; Kaya and Epps, 2004). Because of the negative associations many Americans have about the number 13, for example, American parents might try to avoid this day of birth. Conversely, as already noted, the positive associations most people have about the number 1 may make many parents prefer this birthday number.

Perhaps even more to the point, research on magical thinking shows that people assume that the emotional properties of a stimulus rub off onto things that touch or resemble that stimulus. For example, Rozin et al. (1986) used a new, perfectly clean flyswatter to stir a pitcher of lemonade. To most people, the lemonade suddenly became less desirable. No one thought the flyswatter carried any diseases; they just *associated* the flyswatter with flies. The human aversion to things that resemble disgusting things presumably happens because of the *principle of contagion*. This principle is even stronger for feces than for flyswatters. Making otherwise delicious chocolates shaped like dog feces makes them a lot less desirable. Magical thinking seems to apply to birthdates as well as to shapes and colors. Cialdini et al. (1976) showed that people judged a Russian historical figure (Rasputin, the “Mad Monk”) more favorably than usual when they believed he happened to share their own birthdays. A frequent misperception of research on emotional contagion is that an object must touch another object to be subject to contagion. Although touching matters, simply sharing a symbol with something positive or negative is enough to influence judgment. For example, people like names that merely share letters with their own names (Pelham et al., 2002). Nonetheless, if people think that their children will be holy if born on Christmas—or lovable if born on Valentine's Day—they will probably expect *others* to share their magical assumptions.

## Overview of Analyses

### Analytic Approach

Our basic analytic approach in our early studies was that of Shimizu and Pelham (2008). We assumed that, long ago, the daily distribution of natural human births was random. Thus, across many years, one would predict the same number of births on Christmas Day as on the adjacent days (e.g., December 23). We thus identified the 5-day window centered around each holiday (or day of interest) studied—to see if the number of officially reported births on the exact day of the holiday exceeded the average for the surrounding days. In addition to calculating chi square statistics where appropriate, we calculated Simonsohn's (2011) ratio (R_AE_) statistic. As applied here, this statistic is the frequency of births on a day of interest divided by the average of the frequencies for the neighboring days. This indicator is independent of sample size. A score of 1.43 means, for example, that there were 43% more births on a given day than on the surrounding days. A highly simplified example of data that would yield a value of 1.28 for this statistic (for a 5-day period) is 100, 100, 128, 100, 100.

This statistic works extremely well when averaging data across many years. However, to examine the likelihood of births on a given day (e.g., Christmas Day) in any given year—especially in modern births—there are a couple of other important considerations (as applied, for example, to Studies 5a and 5b). First, in modern U.S. births, there are large day of the week effects. American mothers are about 70% more likely to give birth on a Tuesday than on a Sunday (Pelham, 2021). Second, in any specific month, there are obviously more births on the days of the week that occur five times in that month than on the days of the week that only occur four times in that month. When focusing on a specific holiday in modern data, we always took such complexities into account.

### Datasets Analyzed

For all of the studies in this report, our default was always to use the largest and most representative data set(s) we could locate that would allow a test of our hypothesis. We used smaller data sets (e.g., state level data sets) only when they allowed for specific searches (e.g., exhaustive modern birth records) that were impossible to conduct (i.e., for which there were no data) in larger sources.

For a summary of the datasets used across the studies and the time period covered in each data set, see Table 1. Many of our studies used data from the Social Security Death Index (SSDI), which, at the time of data collection, included 94 million U.S. decedents (who died between 1935 and 2014). According to ancestry.com about 98% of these SSDI decedents died after 1962. Virtually all SSDI records include an exact date of birth. However, when an exact date of birth was unavailable, the SSDI seems to have used the 15th of the month as a place marker (Shimizu and Pelham, 2008). We thus exclude the 15th as a date of birth in analyses based on the SSDI. When using other data sources, we did not need to use this exclusion rule. For Study 1, we chose a 21-year search window because this is the widest window (±10 years from a specific year of birth) for which the ancestry.com search tool allowed searches when we harvested the data. We made births occurring in 1900 the search midpoint for the 21-year search window of Study 1 because virtually all Americans were born at home in 1900 (MacDorman et al., 2012). Notice that we did not define selection windows based on when people *died* but upon when people were *born*—because our hypotheses are about the selection of birth dates.

**Table 1:** Summary of the data sets, websites, and time periods used in this report.

**Study**	**Data Set**	**Website**	**Birth time period**
1	Social Security Death Index	http://search.ancestry.com/search/db.aspx?dbid=3693	1880–2014
2a	Social Security Death Index	http://search.ancestry.com/search/db.aspx?dbid=3693	1880–2002
2b	Virginia Birth Records	ancestry.com's Virginia birth records	1912–2015
3	Social Security Death Index	http://search.ancestry.com/search/db.aspx?dbid=3693	1880–2014
4	Social Security Death Index	http://search.ancestry.com/search/db.aspx?dbid=3693	1880–2014
5a	Virginia Birth Records	ancestry.com's Virginia birth records	1912–2015
5b	US CDC	https://wonder.cdc.gov/natality.html	2016–2019
6a	Virginia Birth Records	ancestry.com's Virginia birth records	1912–2015
6b	Nevada Birth Records	ancestry.com's Nevada birth records	1975–2021

*For reasons explained in the main text, not all possible birth periods were examined in all studies. Further, for SSDI records, 1880 is only an approximate date of the oldest SSDI records but is the oldest date we searched. That is, there were some limited observations older than an 1880 birth date*.

### Overview of Studies

Study 1 assessed whether birthday selection effects exist, whether they are larger for more important holidays, and whether basking in reflected glory (trying to associate one's child with a famous person) is a part of this process. Study 1 examined every official U.S. holiday that was celebrated continuously—and on exactly the same date—each year, between 1890 and 1910. To this list of eight holidays, we added St. Patrick's Day and Columbus Day, to be as inclusive as possible—and to make the list of minor holidays as long as the list of five major public holidays.

Studies 2a and 2b examined changes in birthday selection over 125 years—as births gradually became medically documented at hospitals (leaving less room over time for either false claims or memorial biases).

Study 3 assessed whether there was (a) a larger birthday selection effect for St. Patrick's Day for Irish immigrants and (b) a larger birthday selection effect for *Cinco de Mayo* for Mexican immigrants.

Study 4 examined birthday selection for different calendar days rather than holidays. More specifically, it assessed whether parents long ago more often overclaimed specific calendar days that are more strongly liked in modern samples.

Studies 5a and 5b show that modern American mothers are less likely to give birth on Christmas Day than on the surrounding days. Study 5a examined whether modern Virginia mothers gave birth on or around Christmas Day at rates below those expected by chance. Study 5b assessed what role artificial induction of labor and planned birth by cesarean section play in this Christmas Day aversion.

Finally, Studies 6a and 6b show that many modern American mothers appear to have had labor induction techniques to avoid giving birth on Friday the 13th.

## Study 1: Results and Discussion

**Figure 1:**
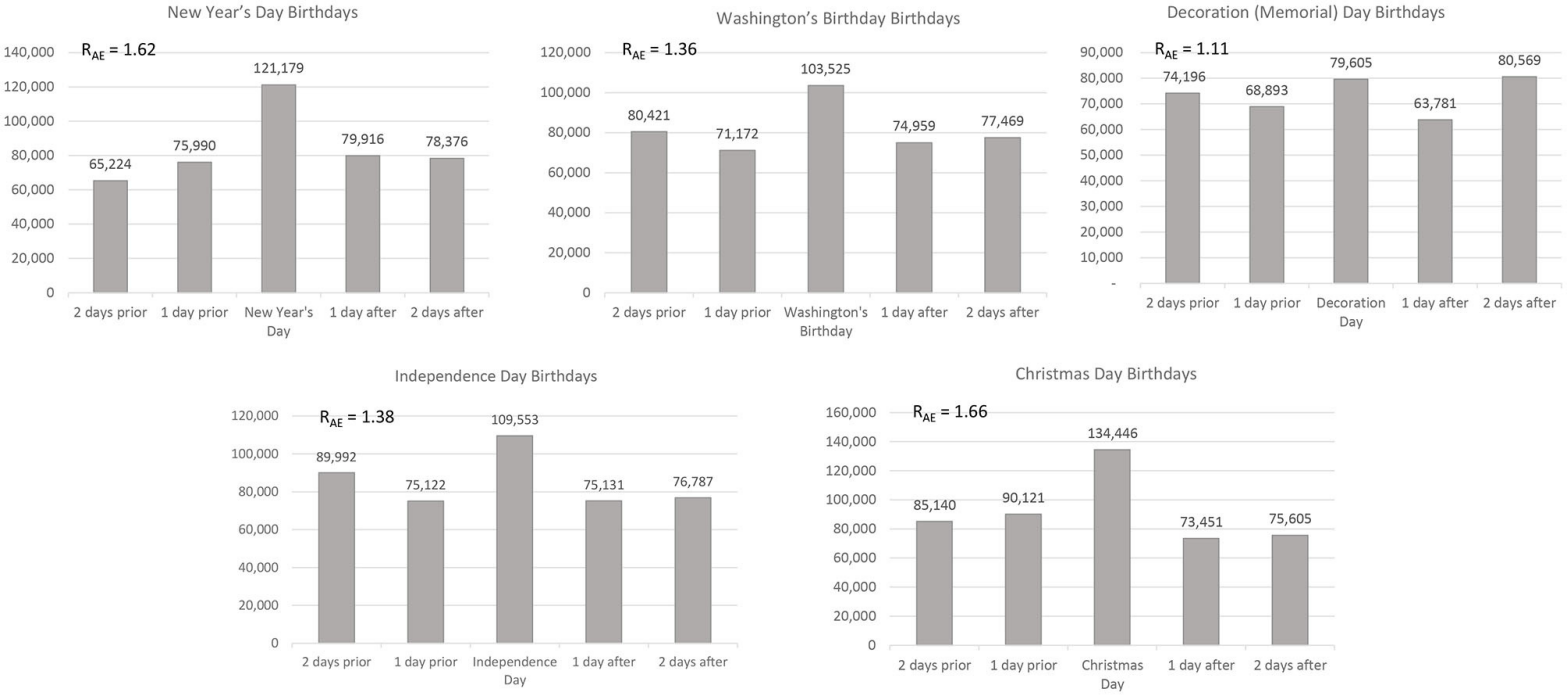
Birthday selection: claiming a major holiday as a birthday for one's child in the U.S. (1890–1910). Two days after Memorial Day was June 1st. It is predictable that parents overclaimed June 1st even more often than they overclaimed Memorial Day because – as we show in in Study 4 – the number 1 is one of the most highly liked of all calendar numbers. On the other hand, supplemental analyses also showed that January 1st was a much more commonly claimed birthday than the first day of any other month of the year.

As shown in Figure 1, parents overclaimed all five of the major holidays as their children's dates of birth. Despite an increase in claimed births on Christmas Eve (which is highly predictable in hindsight, but which worked against predictions), the largest birthday selection effect was for Christmas Day. American parents who gave birth in the 1890–1910 window claimed to have begotten an extra 66% of children on Christmas Day. The effect for New Year's Day (a 62% surplus) was almost as large. The respective biases for George Washington's Birthday and Independence Day were 36 and 38%. The effect for Decoration Day was 11%. Each of these effects was significant, all χ^2^ values > 800, all *p*s < 0.00001.

**Figure 2:**
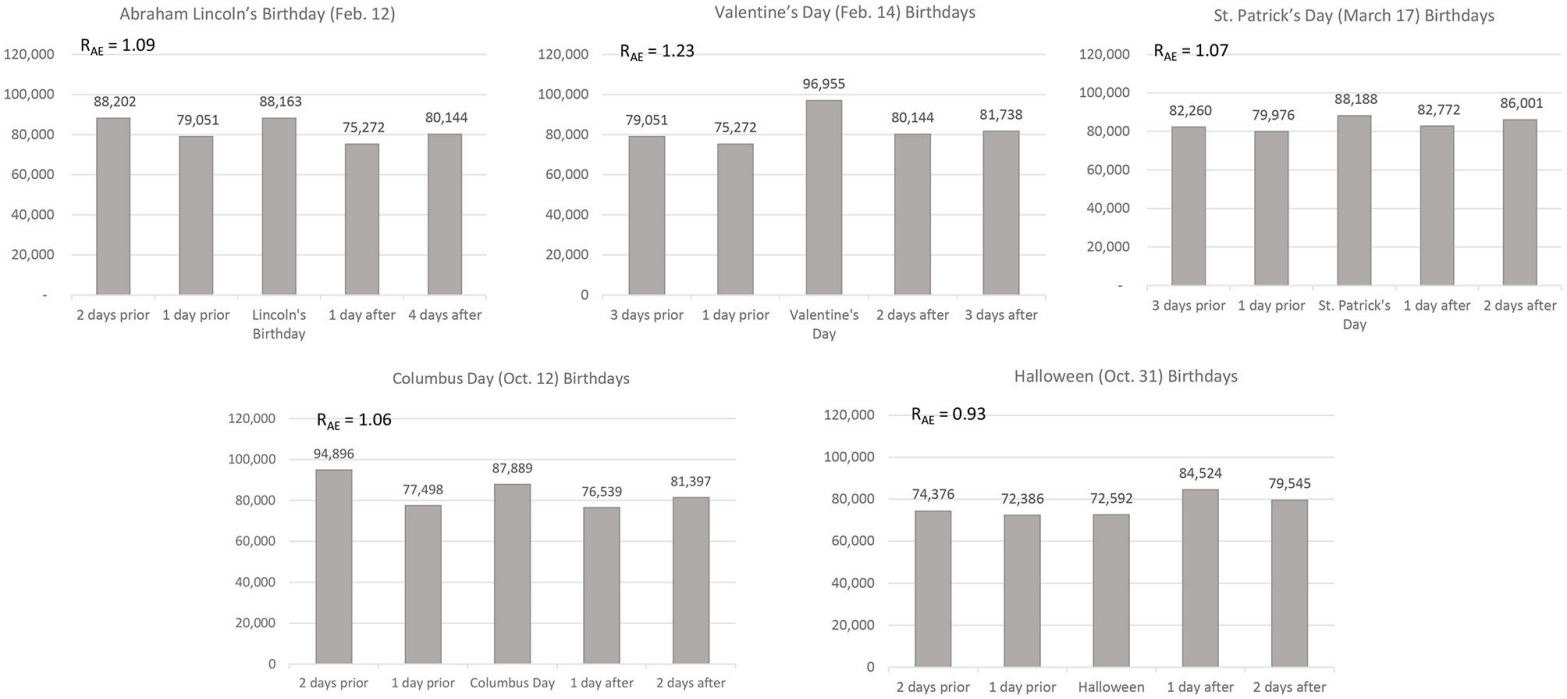
Birthday selection: claiming a minor holiday as a birthday for one's child in the U.S. (1890–1910). When a control day would have been (A) a holiday or (B) the 15th of the month (for which there were not valid data), we chose the next available valid day as a control day. The +9.0% bias for Lincoln's birthday was reduced to +6.9% when ignoring the 13th (a disliked day). The same adjustment for Valentine's Day yielded a +20.7% bias. The −7.0% bias for Halloween became −3.8% when ignoring the first of the month (see Study 4). Finally, the positive biases for both Lincoln's birthday and Columbus Day grow larger when ignoring the highly liked and frequently chosen 10th of the month as a control day.

As summarized in Figure 2, similar but much smaller effects occurred for the minor holidays. In decreasing order of magnitude, the birthday selection effects for the five minor (non-public) holidays included (1) a +23% bias for Valentine's Day, (2) a +9% bias for Lincoln's Birthday, (3) a +7% bias for St. Patrick's Day, and (d) a +6% bias for Columbus Day. There was a modest *negative* bias (−7%) for Halloween, $\chi_{\left(1\right)}^{2}$ = 336.8, *p* < 0.00001. This reversal for Halloween is highly informative. American parents did not simply over-claim memorable dates. Instead, parents strongly overclaimed holidays for which people have highly favorable associations—while steering weakly away from Halloween. Presumably, parents wanted their children to bask in the reflected glory of saints and saviors but did not want them to glow in the questionable company of ghosts and goblins. It is worth adding (a) that we defined holidays as major or minor on an *a priori* basis and (b) that the average effect size for major holidays (42.6% average overclaiming bias) is dramatically larger than that for the minor holidays (a 7.6% averaged bias—and an 11.25% bias when ignoring Halloween). Figure 2 also reveals an important detail that might be easily lost in the analysis of any one specific holiday. For both major and minor holidays, there were usually somewhat fewer claimed births exactly 1 day before and after the holidays than there were exactly 2 days before and after. Christmas Eve (December 24) might seem to be a big exception to this rule, but Christmas Eve is also considered a very special day—if not an official holiday. This suggests, modestly at least, that dates that proved to be “near misses” were overclaimed more often than more “distant misses.” This is also a good point at which to note that—on average—even numbered birthdays were usually liked a bit more than odd numbered birthdays—a finding that closely parallels people's general number liking (The number one, of course, is a strong exception to this rule).

But were these parents really claiming *identities* for their children? Claiming that your child was born on Washington's birthday does not *guarantee* that you were hoping to bask in George's reflected glory. To assess this possibility, we examined naming patterns in a subset of the SSDI data. ancestry.com houses a separate search tool for SSDI data. This tool allows more detailed searches (for 48 million decedents) for those who put in SSDI *claims* (http://search.ancestry.com/search/db.aspx?dbid=60901). This tool includes many more decedent details, including more detailed information on decedents' first and middle names.

We used this tool to see how often parents gave their children the first name (or an obvious variation thereof) of the person being honored in a holiday. As shown in Table 2, for all holidays connected to a specific person, parents disproportionately chose the first name of the famous person as their child's first name. In the case of George Washington, many parents even gave their sons George as a first name *and* Washington as a middle name. Although there were no holidays connected to famous *women*, this did not completely prevent the parents of girls from basking in reflected glory. America produced a surfeit of Georgias on George Washington's birthday—and a plethora of Patricias on St. Patrick's Day. On average, this naming effect was bigger than usual for more distinctive (statistically rare) first names. Unusual first names such as Lincoln, Washington, and Valentine yielded larger effects than extremely common names such as George and Patrick. Thus, the more clearly a name linked a child to the famous person being honored on a holiday, the more likely parents were to claim that name for children ostensibly born on that holiday. An exception to this rule is that Christopher was an *extremely* rare first name back in 1900 but yielded only a very modest effect.

**Table 2:** Likelihood of being named after a famous person as a function of (presumably) being born on a holiday honoring the famous person.

**Holiday**	**−2**	**−1**	**Exact day**	**+1**	**+2**	**R_**AE**_**
Feb 12: Lincoln's Birthday (Abraham)	63	29	129	41	3	3.79
Lincoln's Birthday (Lincoln)	8	10	210	11	5	24.71
Feb 14: Valentine's Day (Valentine)	49	61	581	21	11	16.37
Feb 22: Washington's Birthday (George)	1,267	1,339	5,818	1,228	1,297	4.54
Washington's Birthday (Georgia)	40	26	154	43	37	4.22
Washington's Birthday (Washington)	17	19	341	15	12	21.65
Washington's Birthday (George Washington)	8	11	212	10	4	25.70
March 17: St. Patrick's Day (Patrick)	129	173	648	153	102	4.65
St. Patrick's Day (Patricia)	11	15	181	11	15	13.93
Oct 12: Columbus Day (Christopher)	8	13	30	10	8	3.08
Columbus Day (Columbus)	14	18	21	8	8	1.75
Dec. 25: Christmas Day (Jesus)	18	90	82	26	13	2.23
Christmas Day (Noel)	23	48	221	20	12	8.58

*First (or first and middle) names appear in parenthesis after each holiday. Columns 1–5 are raw frequencies. If a control day fell on another holiday, or on the 15th, we substituted the next available day (prior to or after the usual control day, as appropriate) for that particular control day. For example, for Valentine's Day we substituted Feb. 11 for Feb. 12 (as the control day −2) because Feb. 12 is Abraham Lincoln's Birthday. Virtually all of those who named their children Jesus had Latino surnames. Note that the table values (−2, −1, 0, +1, +2) were not entered into calculations for RAE but merely reflect temporal distance from the target date. Instead, the average of the four surrounding ways was always compared with the crucial day in question*.

Is it problematic for our interpretation of the birthday bias that the overall name frequencies in Table 2 do not match the effect sizes for the biases in birth date frequencies? Is it problematic that Jesus is not a more common name than George? We suggest not, for the simple reason that many other properties of names cause parents to choose or avoid them. For example, almost no non-Latino Americans named their children “Jesus.” Likewise, Columbus is an extremely rare U.S. forename. With such obvious cultural exceptions in mind, the effect sizes were larger for more distinctive names that more obviously connect people to the famous people after whom they were named. The name George, for example, was a very common first name in the U.S. about 120 years ago. It is thus no surprise that choosing this common first name connected children to George Washington less distinctively than did the first name Washington or the highly distinctive first and middle name combination “George Washington.”

Study 1 showed a robust tendency for American parents to select birthdays for their children that would have facilitated an association between their children and a famous person or celebrated event. Of course, these birthday selection and naming effects occurred more than 100 years ago. Do birthday selection effects of any kind exist today? As suggested earlier, some birthday selection effects might even reverse in modern samples. Christmas seems like the most likely example. For these and other reasons, Study 2 assessed changes in the magnitude of birthday selection over time.

## Study 2: Methods

Study 2 used the same SSDI records used in Study 1. For each holiday, we tabulated a birthday selection effect (R_AE_) in every 5-year window centered on 1880–2000 (e.g., 1878–1882, 1883–1887). Thus, the last 5-year window covered was 1998–2002, and the entire study covered 125 years (1878–2002).

## Study 2: Results and Discussion

**Figure 3:**
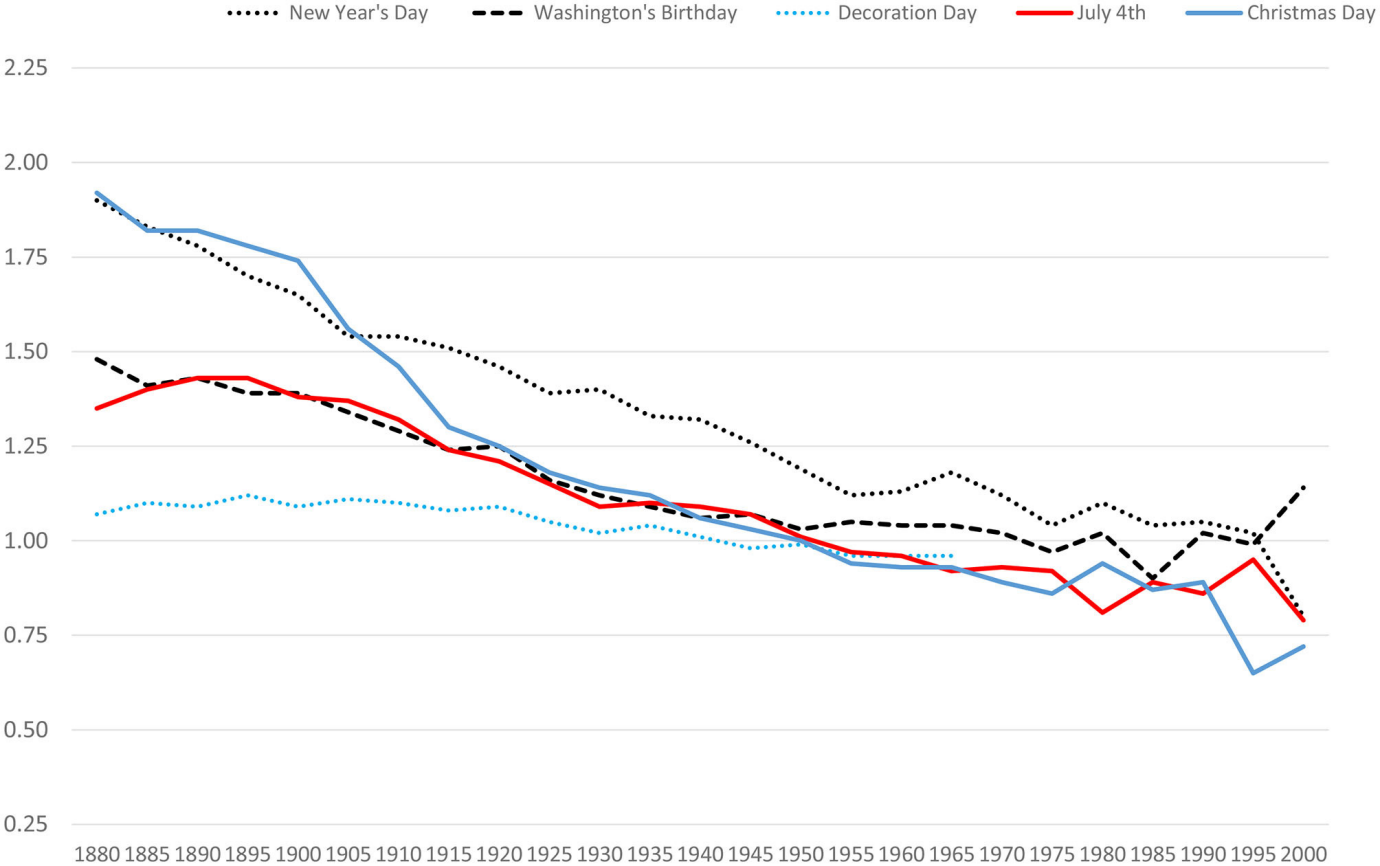
Historical changes in birthday selection for five major U.S. holidays.

Figure 3 summarizes historical changes in birthday selection for major holidays. The most striking finding in this 125-year window is the decrease in the size of birthday selection effects for major holidays. Figure 3 also shows that the decline was sharpest and most immediate for Christmas. By 1950, there was no Christmas Day birthday selection effect. In 1950, by the way, 88% of American women gave birth in hospitals (Wertz and Wertz, 1989). The decline for New Year's Day was also large. But the New Year's Day effect did not disappear until 1995. The bias for Independence Day had disappeared by 1950. Christmas Day, New Year's Day, and Independence Day shared another striking feature. Birthday selection effects for these three holidays did not just disappear in the modern records; they *reversed*. In the 1998–2002 window, all three of these holidays yielded significant *negative* biases (all three χ^2^ values >6.9, all three *p*s < 0.01). On average, only 77% of the expected number of American babies were born on these three holidays. Of course, it is not clear whether modern parents truly avoided these major holidays or whether hospitals steered parents clear of these dates so they could be lightly staffed. We return to this question later in this report.

Although most of these SSDI records are highly representative of the U.S. population, the death records for modern births are not. They exist only for those dying young. Further, the sample sizes become smaller for the modern records (because most people today do not die in childhood). We thus searched *birth* records for both older and modern births. The largest set of such records we could locate were ancestry.com's Virginia birth records, covering 1912–2015. In these records (a) there were robust positive effects for all major holidays in the window centered on 1900 (1890–1910), (b) these effects disappeared or slightly reversed in the window of 1950 (1940–1960), and (c) there was a clear reversal of effects for New Year's Day, Independence Day, and Christmas Day in the latest window (1990–2010). These modern samples were also large (more than 20,000 Virginia births were centered on Christmas, 1990–2010). In these Virginia data, the positive effect for George Washington's birthday weakened over time but never wholly disappeared [RAE = 1.04 for 1990–2010, $\chi_{\left(1\right)}^{2}$ = 9.11, *p* = 0.003]. For holidays that are unlikely to overshadow a child's birthday, modern parents seem to engage in birthday selection. Of course, modern dates of birth are not easily altered. Instead, modern parents gave birth more often than one would expect on George Washington's birthday—and gave birth less often than one would expect on Christmas Day. These decisions were presumably achieved using medical interventions such as induction of labor—an issue we directly examine later in this report.

**Figure 4:**
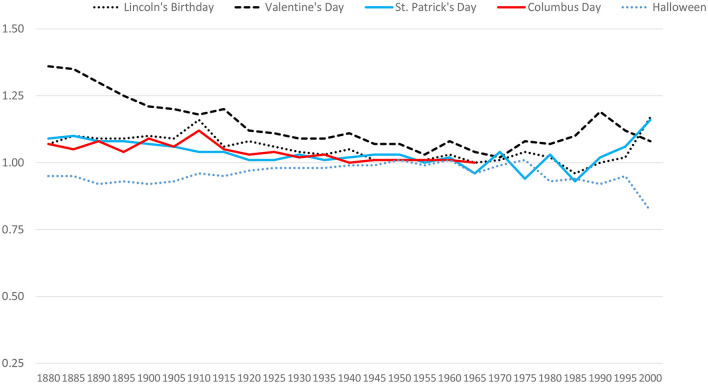
Historical changes in birthday selection for five minor U.S. holidays. Columbus Day data stop in 1965 because beginning in 1971, Columbus Day was celebrated on a different date each year.

In the SSDI data, birthday selection effects for minor holidays grew weaker in the early to mid-1900s, largely disappeared in 1950, and eventually reemerged as positive effects. These findings appear in Figure 4. In these analyses we present Columbus Day only for the window in which it was always celebrated on October 12 (until 1971). As shown in Figure 4, we observed the largest and most robust birthday selection effects for Valentine's Day. In 1880, the effect was a 36% bias (R_AE_ = 1.36). This became as tiny as a 2% bias in 1970, but never evaporated entirely. By the end of the twentieth century the birthday selection bias for Valentine's Day seems to have been about +10%. Lincoln's birthday and St. Patrick's Day also yielded very small biases, hovering around zero in the 1950s and increasing substantially around the year 2000. Halloween showed a negative bias in the early records, disappeared as early as 1940, and re-emerged as a negative bias in the 1980s.

To examine these effects more carefully, we revisited the Virginia birth records. Historical trends for the minor holidays replicated well. For all holidays for which we observed positive overall effects (and for which there were complete data), effect sizes decreased from 1900 to 1950. All three birthday selection effects then re-emerged in 2000–when all holidays showed a positive 4–5% bias (all *p*s < 0.00001). In these Virginia birth data, the effects for Halloween partially replicated. There was no effect in 1950 (R_AE_ = 1.00), and there was a clear negative effect in 2000 (R_AE_ = 0.88, *p* < 0.00001). However, there was no negative effect in Virginia in 1900 (R_AE_ = 1.01).

Study 2 provides insights into the basis of birthday selection effects. The effects in older records seem to be due to motivated memory biases (or blatant confabulation) rather than actual changes in birth frequencies. The data for the minor holidays are probably most telling. The positive biases for Valentine's Day, St. Patrick's Day, and Lincoln's birthday either disappeared completely or dropped to a 2–5% bias when almost all U.S. mothers began giving birth in hospitals. These biases did not emerge again until birth medicalization became commonplace. The mild negative bias for Halloween also disappeared in 1950 and did not re-emerge until the 1980s.

## Study 3: Overview and Methods

Birthday selection is a robust bias, and it varies predictably across holidays and across time. Additional evidence that birthday selection is connected to *identity* would be a connection between birthday selection and ethnicity. In the United States, one of the most important aspects of social identity has long been ethnicity (Hutnik, 1991; Phinney and Devich-Navarro, 1997; Twenge and Crocker, 2002). Holidays vary greatly across ethnic groups. Thus, as already noted, both St. Patrick's Day and *Cinco de Mayo* are (and have long been) perceived very differently by Irish vs. Chicano U.S. immigrants. In Study 3 we took advantage of the fact that the SSDI Claims index includes information about immigration. We compared immigrants born in Mexico with those born in Ireland. We expected to see (a) a larger birthday selection effect for St. Patrick's Day for Irish immigrants and (b) a larger birthday selection effect for *Cinco de Mayo* for Mexican immigrants.

## Study 3: Results and Discussion

**Figure 5:**
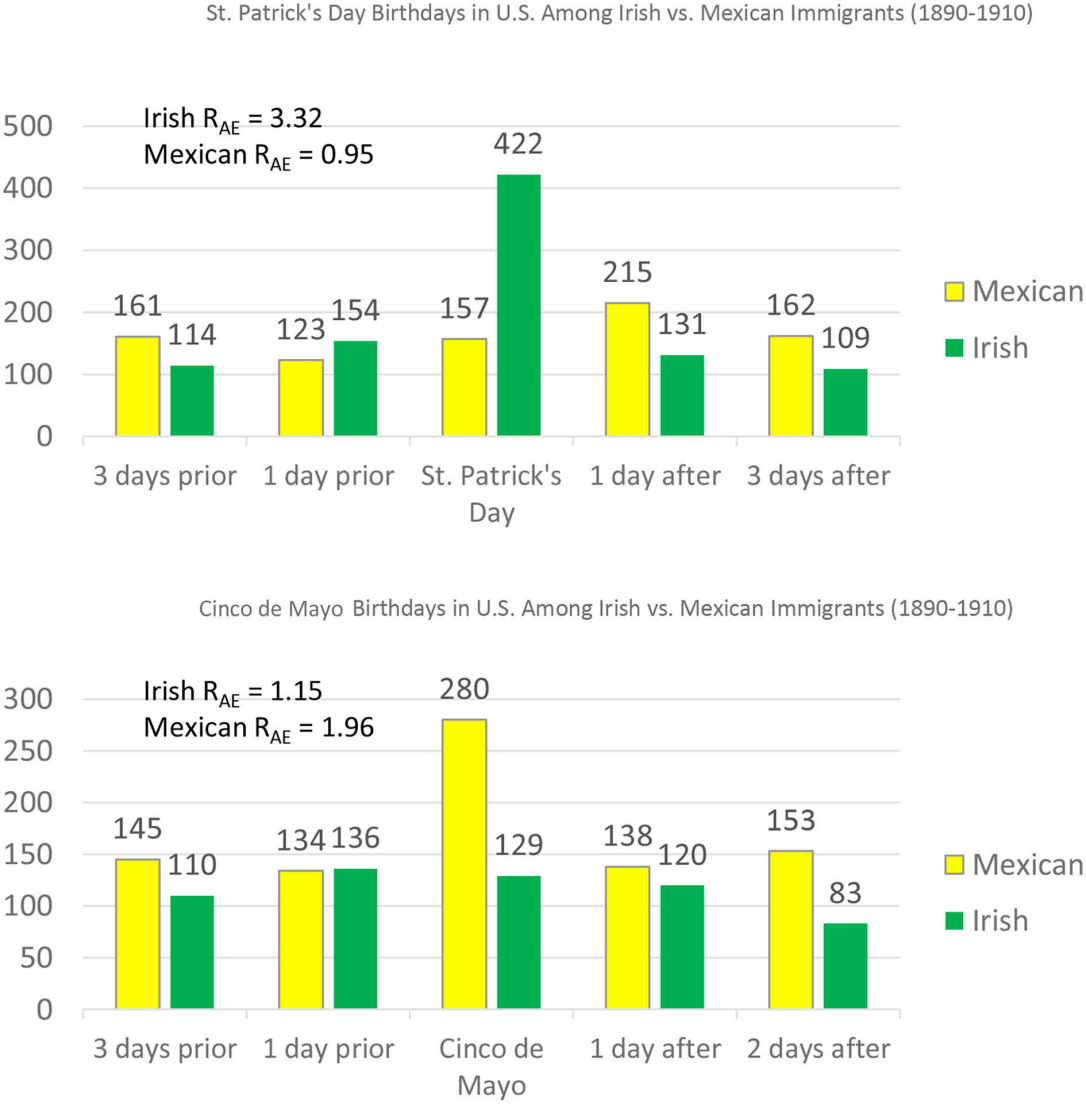
Specificity of birthday selection by ethnicity among U.S. immigrants. We focused on three rather than two days after St. Patrick's Day because March 19th is St. Joseph's Day in Mexico. Further, we focused on three days prior to rather than two days prior to Cinco de Mayo because May 3 is Holy Cross Day in Mexico. Both holidays were overclaimed among Mexican immigrants.

As shown in the top panel of Figure 5, only U.S. immigrants from Ireland showed a St. Patrick's Day birthday selection effect. Irish immigrants reported having St. Patrick's Day birthdays at more than three times the expected rate [R_AE_ = 3.32, $\chi_{\left(1\right)}^{2}$ = 685.24, *p* < 0.00001]. There was no such tendency among Mexican immigrants [R_AE_ = 0.95, $\chi_{\left(1\right)}^{2}$ = 0.41, *p* = 0.522]. As shown in the bottom of Figure 5, this pattern was reversed for *Cinco de Mayo*. Immigrants from Mexico overclaimed Cinco de Mayo—claiming this holiday at almost twice the expected rate [R_AE_ = 1.96, $\chi_{\left(1\right)}^{2}$ = 132.68, *p* < 0.00001]. The tendency in the same direction for Irish immigrants was not significant [R_AE_ = 1.15, $\chi_{\left(1\right)}^{2}$ = 2.50, *p* = 0.114].

Readers concerned about “cherry picking” might be reassured to learn that after testing these *a priori* predictions, we assessed whether Chicanos showed the same pattern for Mexico's Independence Day (September 16th) that they showed for *Cinco de Mayo*. They did [R_AE_ = 1.42, $\chi_{\left(1\right)}^{2}$ = 30.04, *p* < 0.0001], and the Irish immigrants did not [R_AE_ = 1.00, $\chi_{\left(1\right)}^{2}$ = 0.001, *p* =0.975]. Finally, in keeping with the fact that Christmas is popular in both ethnic subcultures, there was a large Christmas Day birthday selection effect in both groups (R_AE_s > 1.50, both *p*s < 0.00001). In a separate report (Shimizu and Pelham, 2021), we address the cross-cultural generality of birthday selection. There is robust evidence for birthday selection effects for cultures across the globe, including highly collectivistic cultures. Birthday selection effects appear to exist across the globe.

## Study 4: Overview and Methods

It appears that wishful thinking can influence something as important as parents' official reports of when their children were born. In Studies 1–3 we focused on holidays. However, other versions of birthday selection may exist. As noted previously, the negative associations many Americans have about the number 13, like the positive associations many have about the number 1 might influence preferences for these birthday numbers. In Study 4, we used the SSDI data set to tabulate reported birth frequencies across the 31 calendar days (ignoring the 15th) in the same window (1890–1910) on which we focused in Study 1. We expected to see a positive association between (a) these 31 reported birth frequencies (correcting for the reduced frequency of the birthday numbers 29–31) and (b) modern Americans' *liking* for the numbers 1–31.

Our data on number liking included all of our existing data from both college students and U.S. adults. More specifically, we used data from (a and b) two independent college student samples in New York state, *n* = 106, 66% female, and *n* = 142, 64% female), (c) one college student sample in Illinois (*n* = 248, 73% female), and a more representative national U.S. adult sample recruited via MTurk (*n* = 290; 51% female). We weighted the four samples equally. Because of variation in the exact liking scales used across samples, we standardized number liking scores within each sample before averaging scores for each number across samples. Relative liking for the 31 numbers was consistent across samples. For example, 13 was disliked in all samples, and 1, 10, and 20 were well-liked in all samples. The reliability of these 31 number ratings was high (α = 0.91). After computing average liking for each number, we assessed the correlation between these liking scores and how many reported births there were on each calendar number day in the 1890–1910 window.

## Study 4: Results and Discussion

**Figure 6:**
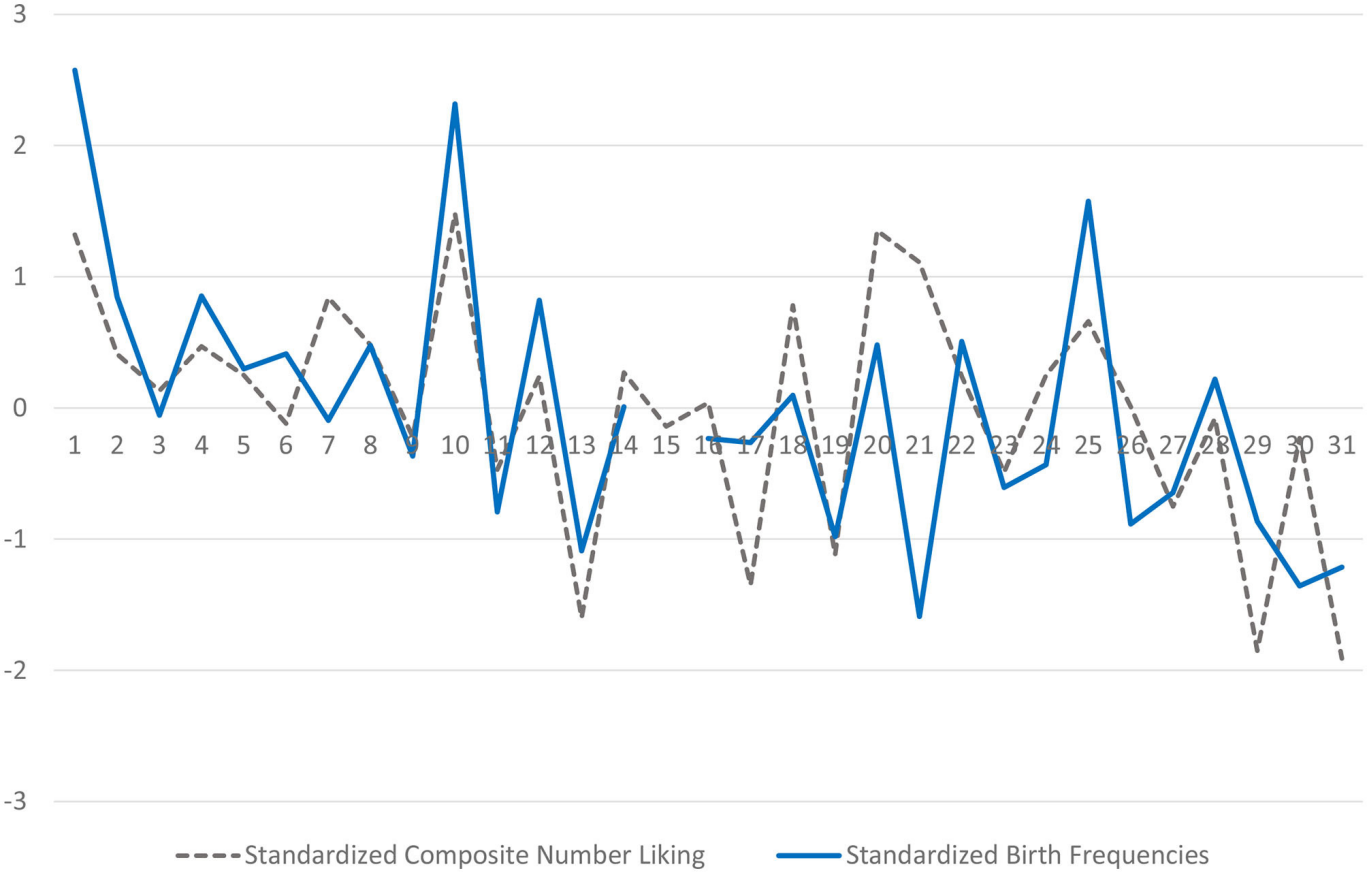
Modern liking for calendar day numbers 1–31 (2013–2015) and SSDI birth frequencies (1890–1910). Day frequencies were corrected mathematically for their actual frequency, taking leap years into account for the 29th of the month. The low birth frequencies for the 29th and 31st of the month thus reflect a true aversion to these dates.

The degree to which modern participants liked the numbers 1–31 predicted variation in the birthday numbers of millions of Americans born around 1900. The composite number liking scores correlated *r*_(28)_ = 0.63, *p* < 0.001, with the day-by-day SSDI birth frequencies. This correlation occurred despite the gap of more than 100 years between the assessment of our predictor and the criterion. Figure 6 clarifies the details behind this correlation. Specifically, it shows exactly how much modern participants liked each calendar number between 1 and 31. It also shows officially reported SSDI birth frequencies for the 30 of the 31 different calendar days for which there were data. It is clear from Figure 6, for example, that modern participants disliked the number 13—and that Americans avoiding claiming 13 as their child's birthday number. Figure 6 also shows that just as participants liked the numbers 1 and 10, American parents overclaimed these two birthday numbers. The only calendar day for which the birth frequency data were notably at odds with the number liking data was the 21st of the month. Modern participants strongly liked the number 21, but the parents of yesteryear steered away from it as a birthday number. A *post hoc* interpretation for this is that most modern Americans (especially college students) associate the number 21 with the legal drinking age. In 1900, this association would not have existed.

Study 4 suggests that, long ago, Americans' positive or negative associations about numbers biased their memories (or at least their claims) of when their children were born. Of course, this interpretation depends on the assumption that collective liking for numbers has been temporally stable over the past century—and across diverse populations. Although this is a difficult assumption to test, the fact that the cross-sample number-liking correlations were highly consistent shows that modern participants who lived in different parts of the U.S. agree about number liking. Further, these number liking ratings (as summarized in Figure 6) have plenty of face validity. For example, the number 13 is a disliked number, and the number 1 is a well-liked number. Birthday selection seems to extend to birth dates that are not official holidays.

## Studies 5A and 5B: Overview and Methods

Studies 1–4 suggest that the strong preferences parents once had for major holidays as birthdays for their children have disappeared—or even reversed. However, even our most recent data stop in the window centered around the year 2000. At this point, there was a substantial bias *against* Christmas Day birthdays. Our explanation for this is that as Christmas became the center of so much collective attention in the past few decades, many parents realized that giving birth on this day had disadvantages. We thus examined the likely origins of modern Christmas Day birth aversions—by seeing what role artificial induction of labor and planned birth by cesarean section play in this modern phenomenon.

The data for Study 5a came from the same Virginia birth records mentioned earlier. The data for Study 5b came from more than 15.3 million U.S. Centers for Disease Control and Prevention (CDC) births that took place between January 1, 2016, and December 31, 2019 (https://wonder.cdc.gov/natality.html). These records include information, for example, about the states in which births took place, and medical information such as gestation period, delivery method, and whether labor was spontaneous or artificially induced. Unfortunately, these records do *not* include exact dates of birth (e.g., Dec. 24), presumably as a precaution to protect patient privacy.

Nonetheless, these records do allow for a mathematical workaround. This is because they include the exact year, month, and *day of the week* of every birth. Consider a concrete example. In 2016, December 1st was a Thursday, which guaranteed that Christmas Eve fell on Saturday and that Christmas Day fell on Sunday. This also meant that there were exactly three Sundays in December that were *not* Christmas Day (December 4, 11, and 18). When averaging all these four Sundays together, one would still expect a deficit of births on Sundays in December of 2016. However, to assess this potential deficit, one must control for two other factors. First, there are large day of the week effects in modern U.S. births. American mothers are about 70% more likely to give birth on a Tuesday than on a Sunday (Pelham, 2021). Second, in any specific month, more births occur on the days of the week that occur five times in that month than on the days of the week that only occur four times in that month. The simplest way to correct for both of these effects is to locate the nearest 31-day *non*-December month that begins on exactly the same day of the week as a December in question—and to compare the daily patterning of births in the two matched months. We located the nearest matching month for each of the four Decembers (2016–2019) for which there were CDC birth records. The respective matching months for December 2016–2019 were (1) March 2018, (2) March 2019, (3) July 2017 and (4) July 2018. This means, for example, that December 25, 2019 and July 25, 2018 were both on a Wednesday.

We then assessed, for example, whether fewer December births than expected occurred on the day of the week that happened to be Christmas Day—relative to the same day of the week in the matched control month. This also allowed us to examine Christmas Eve and the day *after* Christmas. Of course, we were also able to see if any such patterns replicated across the 4 years (2016–2019) for which Christmas Day came on four different days of the week. Finally, we were able to assess whether such patterns were stronger than usual for births that involved labor induction or planned cesarean section than for births that did not involve such medical interventions.

## Studies 5A and 5B: Results

### Results for Exact Day of Birth

**Figure 7:**
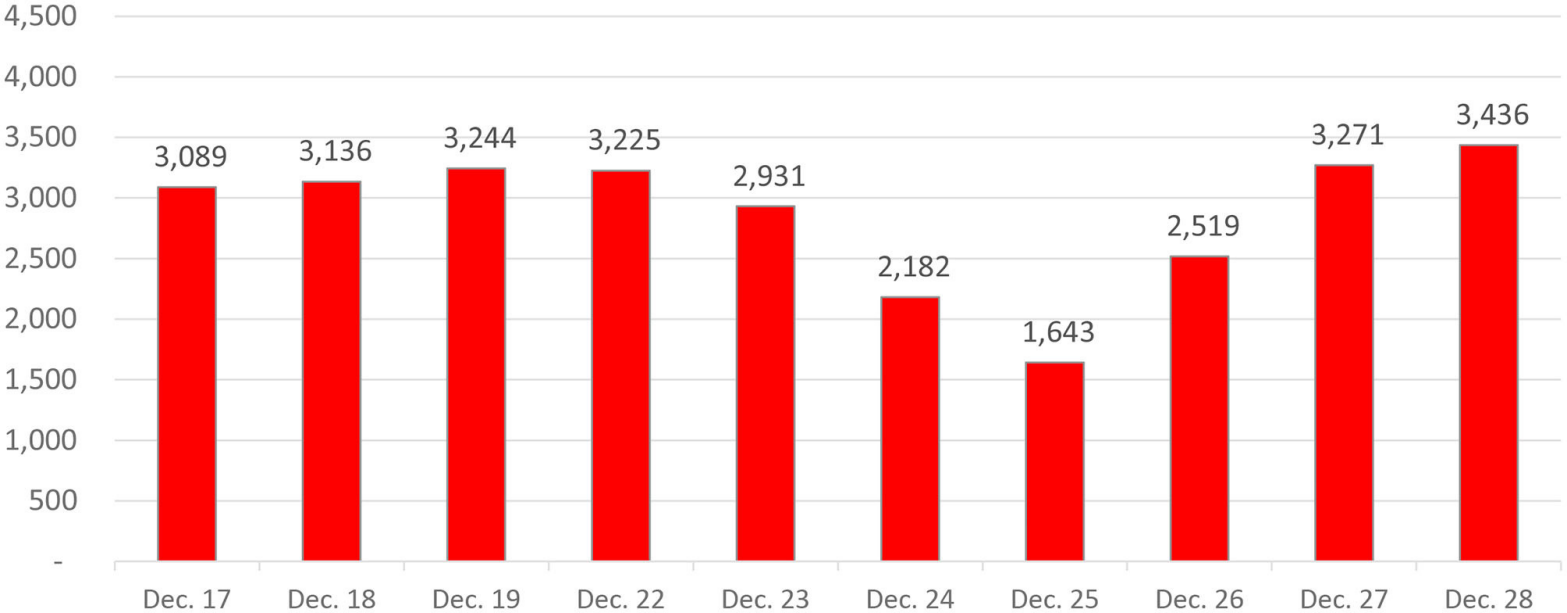
Christmas and near-christmas birthday frequencies in modern (2004–2014) virginia births. Because these frequencies are summed over 11 years (2004–2014), day of the week effects average out.

Do modern births occur less often than naturally expected on or around Christmas Day? They certainly do in Virginia, where day-by-day modern birth records were available. Figure 7 includes exact dates of birth for the 11 most recent complete years (2004–2014) of these Virginia birth records (the complete records end in 2014; the 2015 records proved to be incomplete). As shown in Figure 7, births began to decline slightly as early as December 23, dropped dramatically on Christmas Eve, and dropped even further on Christmas Day. On December 26, births were still well below their pre-Christmas levels. They then showed an abrupt increase on December 27. A comparison of birth frequencies on these 3 days (December 24–26) with the three December days that precede them by exactly a week (December 17–19) reveals that the Christmas window birth deficit is not a day of the week effect. On Christmas Day, there were 52.4% as many births as there were exactly a week prior to Christmas Day (on December 18th). The comparable deficit for Christmas Eve was 70.6%. The deficit remained a substantial 77.7% for the day immediately after Christmas. The only modern statewide U.S. birth records we could locate to assess the replicability of these effects were 1975–2012 birth records for Nevada. Although these records did not allow us to separate births by year, the patterns for all 38 years of records in Nevada were strikingly similar to those observed for Virginia. For example, there were only 65.5% as many Christmas Day births in Nevada as there were births on December 18th. The birthdate depression for Christmas Eve and the day after Christmas also replicated well. Modern births are severely depressed on Christmas Day and for the two neighboring days as well.

### National Results as a Function of Labor Induction

**Figure 8:**
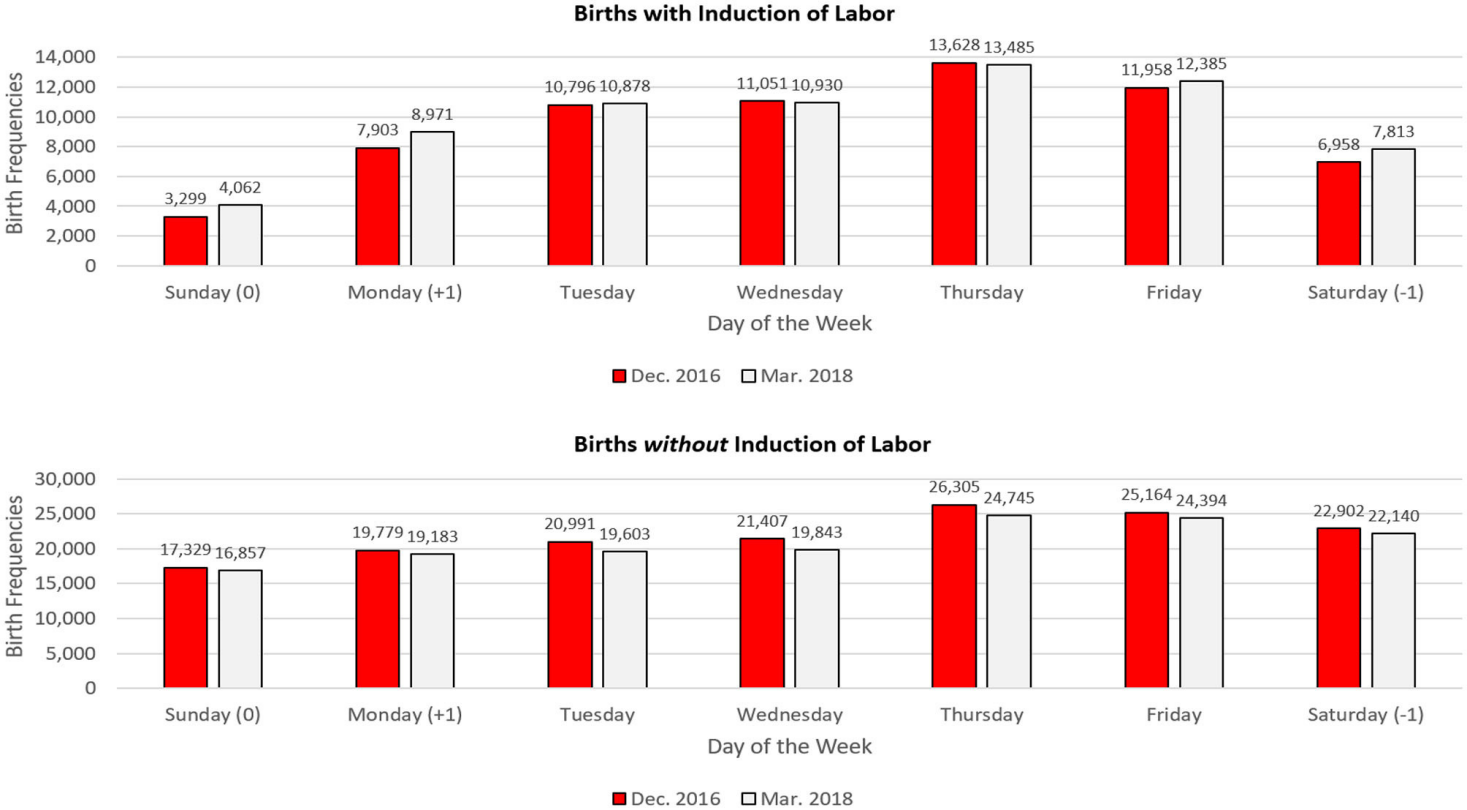
Births in December 2016 and March 2018 by day of the week. The day of the week of Christmas Day (Sunday) is marked with a zero. Saturday (the day of the week of Christmas Eve) is marked −1, and Monday (the day of the week of December 26) is marked +1. Recall that March 2018 has 31 days and began on the same day of the week as December 2016.

Do the birth depression effects observed in Virginia and Nevada apply to the United States as a whole? Do they occur because of artificial induction of labor? The data in Figure 8 suggest that the answer to both questions is yes. The upper half of Figure 8 focuses exclusively on vaginal births for which labor was artificially induced. As shown in the top left corner of Figure 8, there were many fewer induced births on Sundays in December of 2016 (3,299) than on the carefully matched Sundays in March of 2018 (4,062). Further, the data for Saturdays and Mondays in December of 2016 show the same depressions (in attenuated form, of course) that were observed in the date-specific Virginia and Nevada birth records (for Christmas Eve and the day after Christmas). Even after averaging all four Sundays together, there were 81.2% as many induced births on Sundays in December of 2016 as would be expected from the Sunday induced birth totals for March of 2018. After correcting for the fact that March 2018 had 4.4% more induced births than did December 2016, this value became 84.8%. This strong pattern of depressed Christmas births did not hold for vaginal births that were *not* induced. The same thing applies to the Saturday births that corresponded to Christmas Eve and the Monday births that corresponded to the day after Christmas.

**Figure 9:**
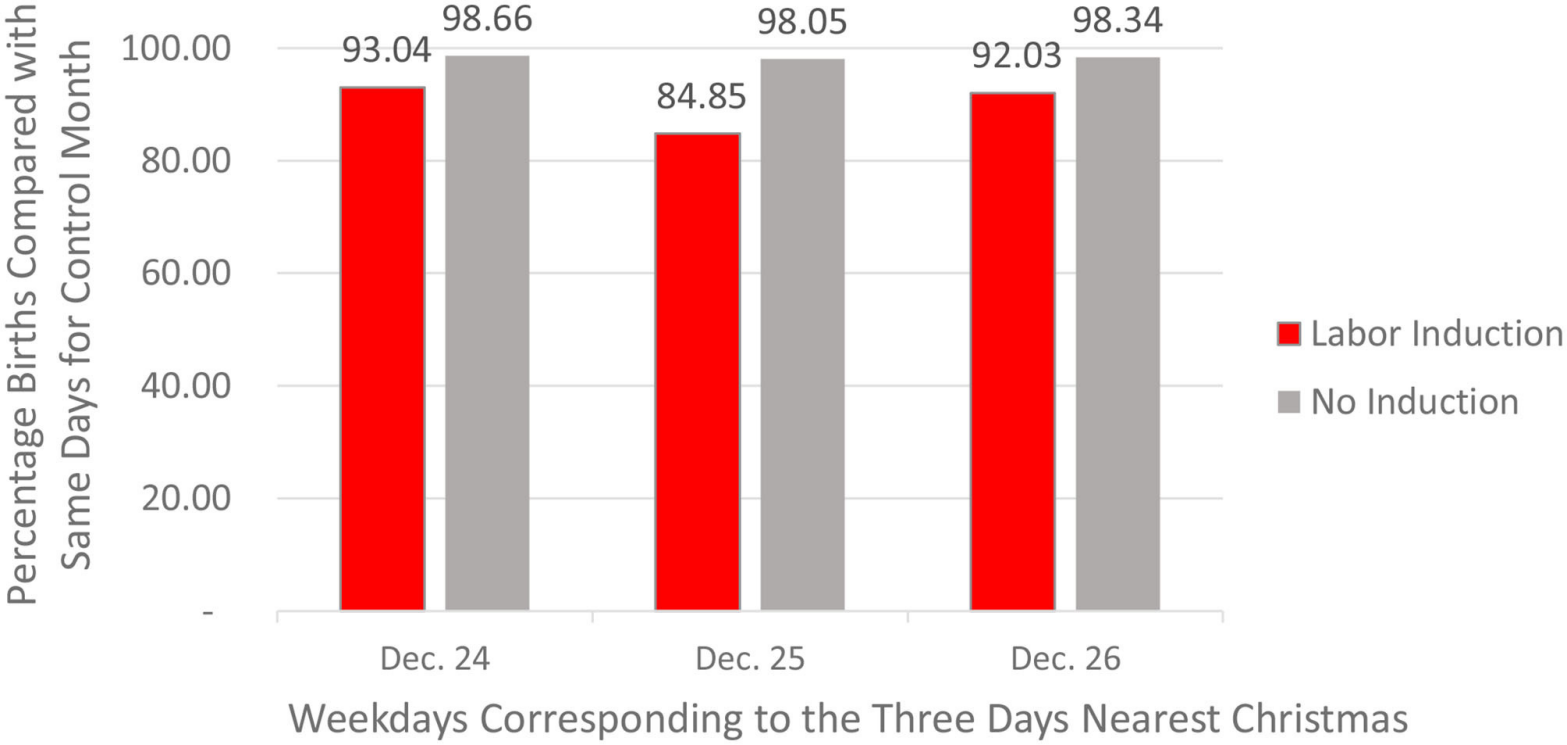
December 2016 birth deficits (compared with same days in control month) for induced and non-induced vaginal births on the days of the week corresponding to December 24, 25, and 26 (CDC Data).

On the other hand, when considering only the non-induced births—and when comparing non-induced births in December of 2016 with non-induced births in March of 2018, there were still Christmas birth suppressions, just much smaller ones. Specifically, there were 98.05% as many *non-medically induced* Sunday births in December of 2016 as one would expect from the data from March 2018. Because of the very large sample sizes, even this small difference was significant, *p* < 0.01. The comparable bias for Christmas Eve was 98.66. For December 26 it was 98.34. Even these very small biases were significant at *p* < 0.05. As shown in Figure 9—which separates induced and non-induced vaginal births for December 24–26 in 2016, the weak effects for non-induced births were real but were much smaller (especially for Christmas Day) than those for induced births. It appears that mothers who do not use modern birth technology may have a real but very weak ability to control their exact dates of birth (e.g., by means of brisk exercise).

**Figure 10:**
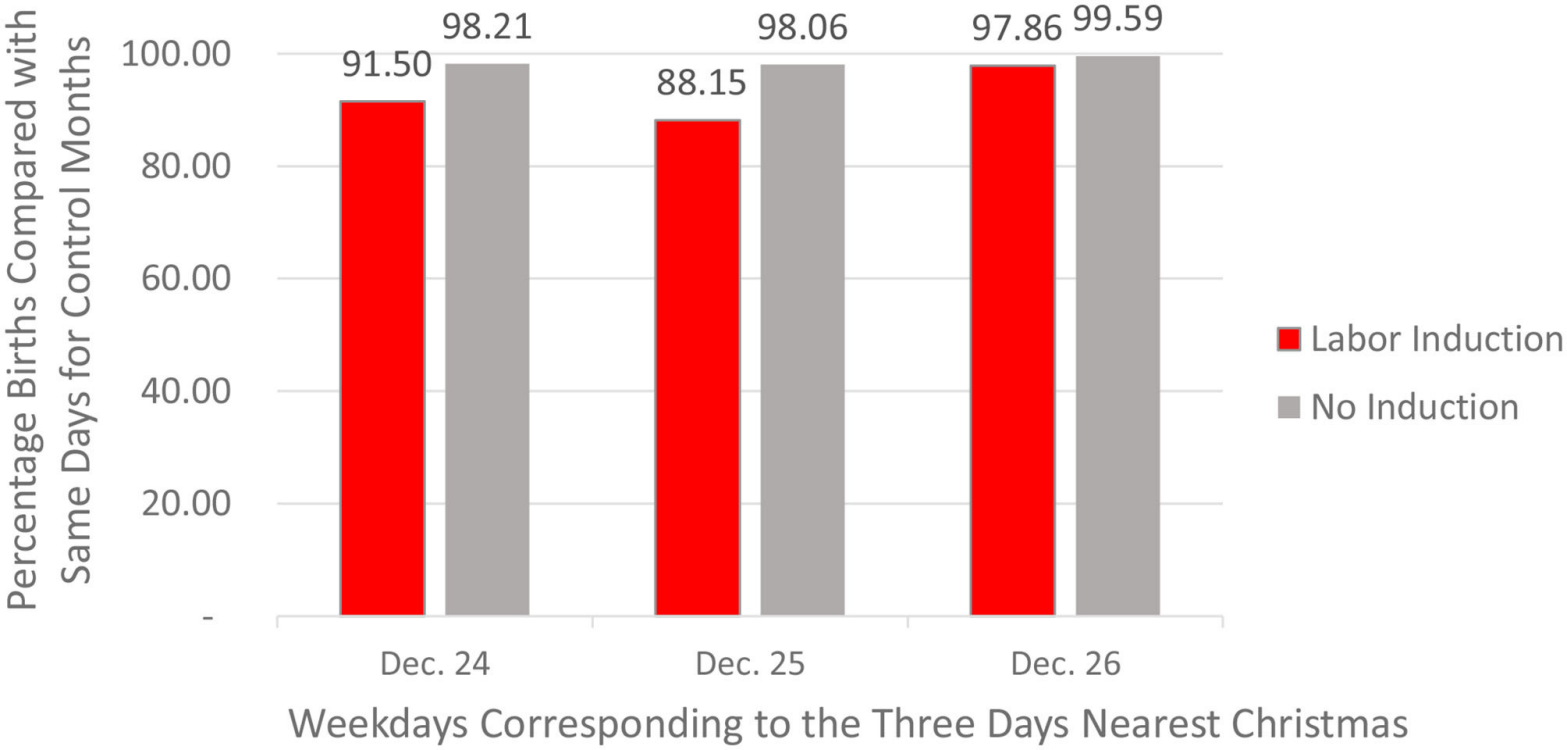
December birth deficits (compared with matched control days) for induced and non-induced vaginal births on the days of the week corresponding to December 24, 25, and 26 (CDC Data, 2016–2019).

These results for 2016 replicated well across the other 3 years for which CDC data existed (CDC birth data for years prior to 2016 do not include information about induction of labor). Taking into account (a) the variations in the total number of births per month across periods, (b) the shifting day of the week that corresponded to Christmas across the 4 years, and (c) the reselection of the temporally closest matching neighbor month across years, there were 82.30% as many Monday births in December of 2017 as for Mondays in the matched month of March *2019*. The comparable statistic for December of 2018 vs. July of 2017 was 93.76%. For December of 2019 vs. July of 2018, the value was 91.69%. Figure 10 shows the mean suppression percentages (weighted equally by year) for the days of the week corresponding to December 24, 25, and 26—averaged across all 4 years for which there were data (2016–2019). The only arguable surprise in these replications was the very weak average suppression effect for births induced on the day of the week corresponding to December 26. On the whole, Figure 10 shows that, as was the case for the December 2016 data, these biases for all 4 years grew much weaker for vaginal births for which there was no medical induction of labor. For births that were not induced, the average Christmas Day deficit was 98.06%, a real but modest bias. Having said that, readers who do the math will notice that a bias of 1.94% missing Christmas births averaged across 4 weeks (three of which are *not* on Christmas) converts to an expectation of no missing births for the three non-Christmas weeks and a likely value of 7.76% missing births (1.94% × 4) on Christmas Day itself.

### National Results as a Function of Planned vs. Unplanned Cesarean Delivery

**Figure 11:**
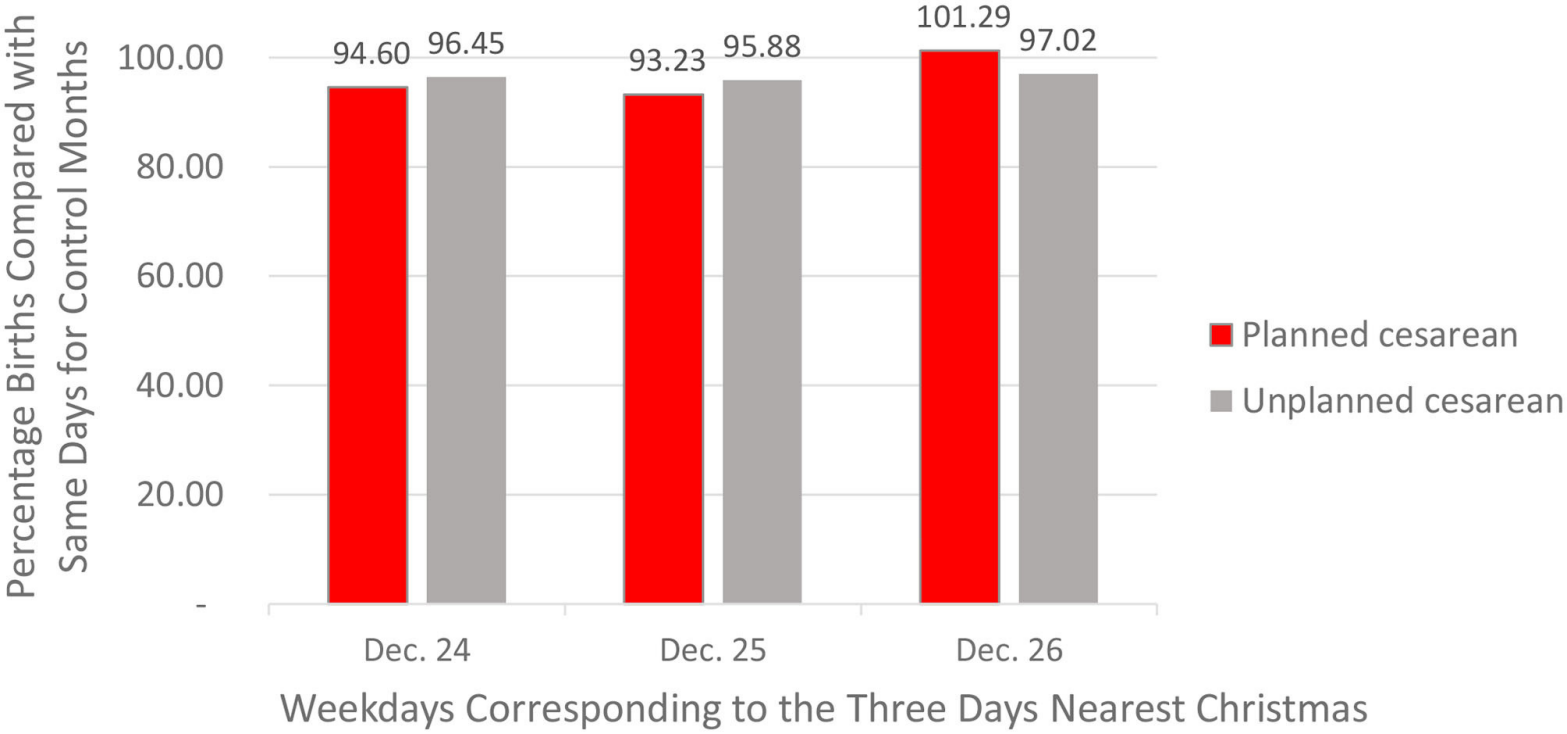
December birth deficits (compared with matched control days) for planned vs. unplanned births by cesarean section on the days of the week corresponding to December 24, 25, and 26 (CDC Data).

Do the patterns of planned vs. unplanned cesarean births parallel those for induced vs. non-induced vaginal births? Recall that it was possible to address this question because the CDC separates cesarean births into those in which (a) there was a trial of labor (*unplanned* cesarean birth) and (b) the cesarean birth was planned in advance (with no attempted labor). The results for cesarean births were a little less consistent than those for vaginal births, but they mainly supported our expectations. In the interest of brevity, we present only the summary findings, averaged across all 4 years. First, as shown in Figure 11, cesarean births in the immediate window of Christmas generally occurred at rates lower than those expected based on the matched control months. Second, when focusing on planned cesarean sections, there was a substantial aversion to planning a cesarean birth on Christmas Day. This was especially true when comparing the days of the week of Christmas Day with the days of the week corresponding to December 26, the only data point for which birth rates were *higher* than expected. In retrospect, this makes sense. After all, those who schedule a cesarean birth immediately after Christmas—like the medical staff who assist them on that day—have a good chance of spending Christmas at home. Figure 11 also shows that the timing of unplanned cesarean births looks much like the timing of births for mothers who gave birth vaginally but did not experience an induction of labor. Aversion rates ranged from 95.88 to 97.02%, a real but modest bias. Because these figures were based on more than 300,000 unplanned cesarean births, even these small biases were significant at *p* < 0.01. These modest depressions could reflect either natural methods of labor induction—or medical labor inductions that led to cesarean births after an unsuccessful trial of labor.

## Study 6A and 6B: Overview and Methods

Study 5b shows that modern parents, medical staff, or both groups working together, use modern birth technology to steer strongly away from births on Christmas Day—and for both Christmas Eve and the day just after Christmas Day as well. But these findings might only reflect the work schedule preferences of hospital staff, including hospital administrators as well as actual health care providers. Do modern parents *themselves* truly have any birth preferences? Even if parents do have such preferences, are they able to convince medical professionals to honor them? To address this question, Study Set 6 returned to the modern Virginia birth records examined previously to examine (a) whether modern parents avoid giving birth on the 13th of the month and (b) whether this tendency is more pronounced than usual when the 13th happens to fall on a Friday. An aversion to giving birth on the 13th would be difficult to attribute wholly to hospital staff preferences. Presumably, very few doctors and nurses demand a day off from work on the 13th of the month.

## Study 6A and 6B: Results and Discussion

Unlike Christmas Day, the 13th of the month occurs every month of the year. A simple way to assess whether modern mothers avoid giving birth on the 13th of the month is thus to compare birth frequencies for the 13th of the month with birth frequencies exactly 1 week before (the 6th) and 1 week after (the 20th). Note that in every month of the year, these three dates always occur with identical frequency and on the same day of the week. Because *Friday* the 13th is considered especially unlucky in the Western Christian tradition, we expected that parents might be more likely to avoid giving birth on *Friday* the 13th than on the 13th in general. The top row of Table 3 includes the complete results for Virginia births. There was a small but significant aversion to giving birth on the 13th of the month, *p* < 0.001. Between 2005 and 2014, there were 17 times when the 13th of the month happened to be a Friday. As shown in the second row of Table 3, Virginia parents clearly avoided Friday the 13th birth dates more fervently than they avoided this birth date on the other 6 days of the week. Row 3 of Table 3 shows the results for the 13th of the month on all the days of the week *other than* Friday. Because of the large sample size, even this small 98% bias was significant, *p* < 0.01.

**Table 3:** Modern Virginia (2005–2014) and Nevada (2002–2011) births on Friday the 13th and on the 13th of the month for all other days of the week.

**6th of month**	**13th of month**	**20th of month**	**Ratio (13th vs. 6th and 20th)**
**Virginia (2005–2014)**			
33,567	32,984	34,572	0.968
**Friday the 6th**	**Friday the 13th**	**Friday the 20th**	**Ratio (13th vs. 6th and 20th)**
5,544	5,030	5,547	0.907
**6th (all other days)**	**13th (all other days)**	**20th (all other days)**	**Ratio (13th vs. 6th and 20th)**
28,023	27,954	29,025	0.980
**Nevada (2002–2011)**			
**6th of month**	**13th of month**	**20th of month**	**Ratio (13th vs. 6th and 20th)**
11,839	11,792	12,203	0.981
**Friday the 6th**	**Friday the 13th**	**Friday the 20th**	**Ratio (13th vs. 6th and 20th)**
1,798	1,662	1,794	0.929
**6th (all other days)**	**13th (all other days)**	**20th (all other days)**	**Ratio (13th vs. 6th and 20th)**
10.041	10,130	10,409	0.991

As shown in the lower half of Table 3, these results replicated pretty well in the much smaller sample of recent Nevada births (which ended in 2011 rather than 2014). The main difference between the results for Nevada vs. Virginia is that in Nevada, the results for days other than Friday the 13th were not significant when taken alone. There are two likely reasons. First, there were many fewer Nevada records than Virginia records. Second, Nevada is a much less religious state than Virginia (Newport, 2009). It is probably no accident that the cultural basis of superstitions based on the number 13 is grounded in Christianity. Ignoring the subtleties of Friday the 13th vs. the other days of the week, the overall aversion to giving birth on the 13th of the month was significant for Nevada as well as Virginia. We could locate no other modern U.S. birth records to further examine this bias in modern births. Astute readers will also notice that it was not possible to use the matching month technique we used for Christmas to assess aversions to the 13th of the month in the CDC birth data. The 25th of June or October is not Christmas, but the 13th of either month is still the 13th. Readers may also recall that the 13th of the month was one of the rarest of all claimed birthdays in the very old records. Modern American parents appear to be very much like their ancestors in that they share the aversion to giving their child a birthday on the 13th of the month. Although the bias we saw in these modern Virginia records was a bit smaller than the bias we observed in the much older versions of these Virginia records, it is worth noting that modern parents were apparently willing and able to convince medical professionals to use modern birth technology to steer away from an undesirable birthday for their child.

## General Discussion

The studies in this report attest to the powerful role intuitive beliefs play in people's preferences for their children's birthdays. They suggest, for example, that basking in reflected glory, superstitious thinking, and the pragmatic goals of health care providers all influence preferences for a real-world outcome, namely a child's date of birth. The finding that birthday selection effects are larger than usual for more important holidays suggests that parents long ago engaged in motivated social cognition or even outright fabrication when recalling their children's dates of birth. This finding, combined with the aversions we have found here and elsewhere for undesirable birthdates, suggests that birthday selection is not simply a matter of memorial accessibility. For example, modern parents clearly avoided giving birth on the 13th of the month. These were actual birthdays rather than remembered birthdays. These findings strongly suggest that parents themselves play at least some role in birthday selection effects. The aversions to certain dates we have observed here and elsewhere suggest that there is much more to birthday selection than simple memorial accessibility.

The naming patterns observed in Study 1 strongly suggest that at least *some* parents try to help their children bask in the reflected glory of famous people—rather than simply misremembering salient dates as their children's dates of birth. Future research should try to dissect impression management processes and magical thinking processes as separate explanations for the effects document here. Given how robust birthday selection effects are, it would be surprising if a single mechanism were responsible for all cases of birth date selection. Many social preferences are overdetermined.

## Limitations of this Research

Many of the studies in this report are open to more than one interpretation. For example, even showing that some parents gave their children names such as “George Washington Johnson” does not guarantee that all parents who chose this birthday for their children were trying to bask in reflected glory. Likewise, most of these studies cannot tell us exactly which parents falsely claimed desirable birthdays and which ones reported birthdays accurately. Further, we assume that more than one psychological mechanism is likely to be at the root of the preferences documented here. Along similar lines, we did not present any evidence that magical thinking played a role in any of these preferences. To address this concern, Pelham and von Hippel (2021) directly asked both parents and college students whether they endorsed “magical” beliefs about birthdays and holidays. These preliminary studies show that people clearly believe that others expect children to possess the traits associated with certain holidays. For example, people report that they believe most Americans think that a child born on Christmas Day will be perceived as “holy” and “generous” —whereas a child born on September 11th will be perceived as “evil” and “Anti-American.” Respondents even concede that they themselves endorse such beliefs—although to a weaker extent than they think such beliefs are endorsed by others. Magical thinking appears to be at least part of the reason for the preferences documented here. But this reinforces the fact that the present studies could not definitively identify the exact mechanisms responsible for the birthday selection effect.

Future research might also assess whether birthday selection in our older data is grounded in (a) motivated memory biases (ranging from self-deception to “judgment calls” that happen when children are born in very close proximity to midnight), or (b) consciously calculated fabrications. Illusory beliefs that portray us and those we love in a favorable light appear to be both more satisfying and more convincing to others when we truly believe them ourselves (Murray et al., 1996; Von Hippel and Trivers, 2011). But it is surely reasonable to assume that *some* of these parents consciously claimed a desirable birthday for their children. And if some of these parents lied about their children's birth dates, one must ask *why* they did so. Our findings suggest that at least some of these parents may have felt their children would benefit from basking in the reflected glory of a famous person or event. It is less clear that an obvious fabrication of a child's birth date could allow parents *themselves* to conclude that their child was especially holy. Magical thinking probably works best among people who personally buy into the relevant association. However, as Von Hippel and Trivers (2011) have argued, the line between self- and other deception is much fuzzier than most people assume. People who are able to convince themselves that a fabrication is true may well have an easier than average time convincing others.

Do our results reflect “cherry picking”? When researchers are able to study entire populations rather than convenience samples, and when they are able to examine every possible operationalization of a variable (as one can do with U.S. holidays but not with variables such as “self-esteem” or “cognitive dissonance”), concerns about “cherry picking” and “researcher degrees of freedom” are largely ruled out. Of course, one can always open the conceptual net—by examining the cross-cultural generality of an effect, for example. This is exactly what some members of this research team are currently doing. Whatever one's perspective, the findings reported here pave the way for critics who might wish to disconfirm our hypotheses. For example, the Social Security Death records examined here are freely available at ancestry.com. Thus, it would be possible to see how robust any one of these holiday biases is across the 50 U.S. states—or to see if these biases vary with cultural variables such as collectivism (see Vandello and Cohen, 1999). Our initial analyses suggest that cultural variables do matter. Both the pro-Christmas bias of yesteryear and the preference for July 4th birthdays were stronger than average in more collectivistic U.S. states. The Christmas Day preference was also much stronger than average in more religious U.S. states. As a final example, researchers could manipulate well-studied self-concept motives in the lab (e.g., using self-affirmation vs. self-concept threat manipulations) to examine the effects of such manipulations on the birthday preferences examined here.

One surprising aspect of our findings in Study 4 is that birthday number effects (e.g., the 11th vs. the 12th of the month) were so large in the 1890–1910 window that they introduced some noise into the assessment of some of the 10 specific holidays we examined in Study 1. In principle, one could ipsatize (i.e., proportionalize) the 31 days of the month during a given historical period to reduce noise when creating effect sizes for the holidays in the same exact window. Of course, these ipsatized scores would need to be calculated separately for different temporal windows, making this task a bit more complex than it would be otherwise. Having said all this, our supplemental analyses (e.g., those comparing claimed birthdays on the 25th of December vs. the 25th of other months) do make it very clear that holiday effects are not merely calendar day effects—or day of the week effects—in disguise. It is also possible that different psychological mechanisms underly the subtle preferences for certain calendar days and the preferences for specific holidays. It is not clear, for example, whether any parents would ever expect their children to bask in the glow of the likable number 14.

Of course, arguments such as these rest on the assumption that a person's birthday can be a part of a person's identity. Is this true? Research suggests so. Most people strongly like their birthday numbers (Kitayama and Karasawa, 1997). Further, many researchers have incorporated liking for one's birthday numbers into measures of implicit self-esteem (DeHart et al., 2006). In principle, any letter, number, or symbol that is associated with a person can become a part of a person's identity. Former professional athletes Reggie Jackson and Wayne Gretsky even incorporated their uniform numbers into their signatures (Armstrong, 1986). Of course, fans of famous athletes often advertise their psychological connection to such athletes—and to the teams for which the athletes play—by wearing copies of the jerseys of famous athletes. Symbols and events that are connected to us and to the groups to which we belong truly become a part of us. Finally, even if most people were *not* highly invested in their birthdays, those who believe that their birthday is special—because it doubles as a widely adored holiday—might be expected to identify more strongly than average with their birthdates.

These findings attest to the power and pervasiveness of both identity and magical thinking. We have long known that human beings care deeply about their identities (James, 1890; Cooley, 1902; Mead, 1934), including the ethnic, religious, and cultural groups to which they belong. The present findings suggest that parents are keenly aware of the subtle ways in which people can derive a sense of value or worth by merely sharing a birthday with a person who is deeply valued or respected by most others. Likewise, taken together, these studies strongly suggest that magical thinking is alive and well in the distortion and creation of children's dates of birth. For well over a century, U.S. parents have apparently gone to great lengths to create happy birthdays for their children.

## Data Availability Statement

The raw data supporting the conclusions of this article will be made available by the authors, without undue reservation.

## Ethics Statement

The studies involving human participants were reviewed and approved by Montgomery College IRB (required for only one of six studies) most studies use public data. The patients/participants provided their written informed consent to participate in this study.

## Author Contributions

BP did the majority of the writing and harvested all of the data—with the exceptions that TD, MS, and HH all independently collected name letter liking data. All authors actively edited multiple drafts of the report and made numerous suggestions for the design of Studies 4–6. CH and WH also suggested ways to separate basking in reflected glory and magical thinking as likely drivers of these effects. BP conducted all data analyses. All authors contributed to the article and approved the submitted version.

## Conflict of Interest

The authors declare that the research was conducted in the absence of any commercial or financial relationships that could be construed as a potential conflict of interest.

## Publisher's Note

All claims expressed in this article are solely those of the authors and do not necessarily represent those of their affiliated organizations, or those of the publisher, the editors and the reviewers. Any product that may be evaluated in this article, or claim that may be made by its manufacturer, is not guaranteed or endorsed by the publisher.

## Appendix A: The Five Major and Five Minor U.S. Holidays Examined in This Report

### Major Holidays

#### New Year's Day

This holiday is celebrated annually on January 1st to mark the arrival of a new calendar year. Many celebrate it in parties that begin on the eve of this date. It became an official U.S. holiday in 1870.

#### George Washington's Birthday

This holiday (February 22) honors the first U.S. president. It became official in 1880. In 1971, it became *President's Day* and began being observed on the third Monday of every February.

#### Decoration Day (aka, Memorial Day)

This holiday honors those who died in U.S. military service. Between 1868 and 1970, it was celebrated on May 30. In 1971, it became the final Monday of May of each year.

#### Independence Day (aka “July 4th”)

This holiday marks the signing of the U.S. Declaration of Independence, that is, the nation's birthday. It became an official holiday in 1870. Today, it is also a busy day in many hospital emergency rooms. To this day, some Britons are still not very keen on this holiday.

#### Christmas Day

This religious holiday became official in the United States in 1870. It has long been celebrated in nations with a Western European influence. It was apparently created to help Christians survive long, dark European winters. It eventually became a way to market Coca-Cola and sell toys and electronics.

### Minor Holidays

#### Lincoln's Birthday

This unofficial holiday honors the 15^th^ U.S. president, whose birthday was February 12. Although never an official U.S. holiday it became an influence on the eventual creation of President's Day.

#### Saint Valentines' Day

This holiday has its ancient roots in several Christian martyrs named Valentinus. It apparently became associated with romance at the time of Geoffrey Chaucer. Romantic partners exchange love notes and romantic gifts on this holiday. It has been widely celebrated in the U.S. since the mid-1800s.

#### Saint Patrick's Day

This unofficial holiday honors the Irish Saint Patrick, who brought Christianity to Ireland in the fourth century and is credited with ridding Ireland of all snakes. Waves of Irish immigrants in the 1800s helped popularize this holiday. In some snake populations, it is considered a day of mourning.

#### Columbus Day

The official status of this holiday to mark Columbus's arrival in the New World has varied widely across time and U.S. states, but was traditionally observed on October 12. In 1971, it became an official U.S. holiday, but the date of observance (the second Monday of October) began to vary annually.

#### Halloween

Halloween (October 31) originated in Celtic harvest festivals. By the late 1800s in the United States, it had become widely known. Children have long gone “trick or treating” on this spooky holiday. In the past few decades, many U.S. adults have begun to celebrate this holiday. Today, this unofficial U.S. holiday is probably best summarized by a popular warning: “Cape does not enable user to fly.”
